# Transcriptomic Profiling of Oxidative Stress-Associated Immune and Apoptotic Signatures and In Silico Prediction of Therapeutic Candidates for Sjögren’s Syndrome Dry Eye

**DOI:** 10.3390/biomedicines14102277

**Published:** 2026-10-08

**Authors:** Jiao Wang, Qiping Qing, Jing Zhang, Siyuan Shi, Xue Yang, Xiaoyu Cai, Zihuan Wang, Weiqian Jiang, Qi Zhang

**Affiliations:** 1 Dry Eye Diagnosis and Treatment Center, Chongqing Key Laboratory for the Prevention and Treatment of Major Blinding Eye Diseases, Department of Ophthalmology, Chongqing Branch (Municipality Division) of National Clinical Research Centre for Ocular Diseases, The First Affiliated Hospital of Chongqing Medical University, Chongqing 400016, China; wangjiaocqyk@163.com (J.W.);; 2 The Second Affiliated Hospital of Chongqing Medical University, Chongqing 400010, China

**Keywords:** Sjögren’s syndrome dry eye, oxidative stress, bioinformatics analysis, molecular dynamics simulation, natural antioxidants

## Abstract

**Background/Objectives:** Sjögren’s syndrome (SS) is a systemic autoimmune disease characterized by lacrimal and salivary gland dysfunction, leading to dry eye and dry mouth. This study aimed to explore the potential associations between oxidative stress-mediated immune and apoptotic responses in Sjögren’s syndrome dry eye (SSDE) and to computationally predict theoretical interacting candidates. **Methods:** We enrolled 16 SSDE patients and 15 healthy controls to assess key clinical parameters. The study utilized conjunctival impression cytology, RNA-seq, and bioinformatics to identify exploratory transcriptomic signatures. Gene expression levels were then assessed using real-time quantitative PCR (RT-qPCR). To provide supportive cross-species biological context regarding local oxidative stress status in vivo, malondialdehyde (MDA) levels were quantified in the extraorbital lacrimal glands of an SSDE murine model (NOD mice vs. BALB/c controls, *n* = 6/group). Furthermore, molecular docking and 100 ns molecular dynamics simulations were conducted to computationally explore potential pharmacological interventions. **Results:** Exploratory global transcriptomic analysis indicated that differentially expressed genes (DEGs) were prominently enriched in immune responses, inflammatory signaling, and apoptotic pathways, aiding the identification of a candidate transcriptomic signature of oxidative stress-related DEGs. Preliminary immune infiltration profiling suggested that the abundance levels of CD4+ T cells and eosinophils were positively correlated, while M1 and M2 macrophages were negatively correlated. Exploratory ROC analysis suggested the potential discriminative value (AUC 0.7–0.9) for key disease-associated genes, notably *CDKN1A* and *BAX* (r = 0.883). RT-qPCR results demonstrated that the transcription levels of *CDKN1A*, *BAX*, *TGFBR1*, and *S100A9* were significantly higher in the SSDE group compared to the healthy control group (*p* < 0.05). Importantly, in vivo biochemical assays provided supportive cross-species evidence of lipid peroxidation, demonstrating a significant elevation in MDA levels in the lacrimal glands of NOD mice compared to controls (*p* < 0.01). Furthermore, in silico molecular simulations suggested that natural antioxidants, such as ursolic acid and andrographolide, exhibited favorable predicted binding conformations within the active pockets of CDKN1A and BAX. **Conclusions:** Supported by exploratory transcriptomic profiling and supportive in vivo biochemical data, our findings highlight an exploratory molecular association between oxidative stress and an intimately coupled immune–apoptotic axis in SSDE pathogenesis. The computational structural evaluation of ursolic acid and andrographolide provides a hypothesis-generating foundation for future in vitro and in vivo pharmacological investigations.

## 1. Introduction

Sjögren’s syndrome (SS) is a chronic autoimmune disorder that primarily affects middle-aged women, characterized by the lymphocytic infiltration of exocrine glands leading to dry eyes and dry mouth [1,2]. Patients frequently experience symptoms like dry eyes, often accompanied by stinging or burning sensations. In more severe cases, these symptoms can result in considerable visual impairment, a condition known as Sjögren’s syndrome dry eye (SSDE) [3]. Diagnosing SSDE remains challenging due to frequent discrepancies between subjective symptoms and objective clinical signs, as well as the lack of reliable molecular biomarkers reflecting the profound ocular surface stress state [4]. Consequently, current clinical interventions often fall short of addressing the underlying autoimmune pathologies and fail to provide satisfactory symptom relief across diverse patient populations.

The conjunctiva acts as a protective layer for the eyes, covering the inner eyelids and the front surface of the eyeball, fulfilling essential functions in lubrication, immune defense, and maintaining epithelial integrity [5]. In SS, the dysregulation of the systemic immune system triggers immune cell infiltration into the conjunctiva, leading to keratinization, epithelial cell atrophy, goblet cell dysfunction, and reduced mucin secretion [6]. These pathological alterations contribute to a vicious cycle driven by localized oxidative stress and inflammation that exacerbates ocular surface damage. Current diagnostic techniques, including conjunctival staining and conjunctival impression cytology (CIC), are employed to assess epithelial cell integrity and goblet cell density [7]. However, the non-invasive CIC technique yields a strictly constrained cellular mass, which has historically restricted comprehensive multi-omic and deep protein-level profiling. Nonetheless, reliable biomarkers for SSDE are still inadequately defined, and the molecular mechanisms underlying conjunctival damage remain only partially elucidated. Furthermore, standard therapeutic approaches primarily rely on artificial tears and anti-inflammatory agents [8], which do not consistently alter disease progression or provide universal relief. Thus, there is a pressing need for exploratory diagnostic biomarkers and innovative therapeutic interventions. To address this gap, this study aims to explore the oxidative stress-mediated transcriptomic landscape of SSDE.

Oxidative stress arises due to an imbalance between antioxidant defenses and pro-oxidant activity [9]. In healthy ocular environments, a state of redox balance is preserved; however, in SSDE, a reduced antioxidant capacity (as evidenced by decreased activity of superoxide dismutase [SOD] and glutathione peroxidase [GPX4]) coincides with elevated levels of lipid peroxidation markers (such as malondialdehyde [MDA]) found in tears and conjunctival tissues [10]. These biochemical changes correlate with the severity of symptoms, suggesting the potential involvement of oxidative stress in the pathogenesis of SSDE [11]. Similar redox imbalances, marked by excessive reactive oxygen species (ROS) production and depleted glutathione levels, have been documented in autoimmune diseases such as rheumatoid arthritis (RA) and systemic lupus erythematosus (SLE) [12]. Despite these established macroscopic insights, the precise molecular networks bridging oxidative stress to downstream immune-mediated and apoptotic damage in the SSDE conjunctiva remain incompletely elucidated.

To address the difficulties in obtaining SSDE-specific sequencing data caused by the inherently restricted cellular yield of non-invasive clinical sampling techniques [11], this research integrates whole-transcriptome RNA sequencing of clinical conjunctival specimens with robust bioinformatics pipelines to identify candidate transcriptomic signatures linked to oxidative stress. Through molecular docking and molecular dynamics (MD) simulations, our objective is to computationally evaluate potential theoretical interactions between candidate bioactive phytochemicals and key target proteins, thereby establishing an exploratory in silico foundation for future translational and pharmacological validation.

## 2. Materials and Methods

### 2.1. Patient Inclusion and Informed Consent

This investigation included a cohort of 16 patients diagnosed with SSDE, alongside 15 healthy control subjects matched for age and sex, recruited from the Department of Ophthalmology at the First Affiliated Hospital of Chongqing Medical University. The diagnosis of SS was established according to the 2016 American College of Rheumatology/European League Against Rheumatism (ACR/EULAR) classification criteria [2]. The diagnosis of SSDE required patients to concurrently fulfill both the 2016 ACR/EULAR classification criteria for SS and the standard diagnostic criteria for dry eye outlined in the Tear Film & Ocular Surface Society (TFOS) Dry Eye Workshop (DEWS) II report [13], with detailed clinical evaluation parameters summarized in Table S1. The TFOS DEWS II diagnostic methodology is based on the following four clinical parameters: the Ocular Surface Disease Index (OSDI), tear film break-up time (TBUT), Schirmer I test, and corneal fluorescein staining (CFS).

Individuals with a history of ocular surgery, trauma, contact lens usage, or any autoimmune comorbidities were excluded from the study. Ethical approval was obtained from the institutional review board (Approval No. 2023-153), and written informed consent was obtained from all participants in strict accordance with the Declaration of Helsinki.

### 2.2. Conjunctival Impression Cytology (CIC)

After administering local anesthesia to the ocular surface using 0.5% oxybuprocaine hydrochloride, a cellulose acetate filter strip (NC Transfer Membrane, BioSharp, Hefei, China), which measured 6.2 mm in diameter with a pore size of 0.45 μm, was positioned on the inferior nasal conjunctiva near the limbus with the opaque side facing downward. The filter strip was gently applied to the conjunctiva using tweezers for a duration of 2–3 s before being carefully detached. Subsequently, it was placed immediately into an Eppendorf tube and stored at −80 °C. Crucially, owing to the extremely restricted cellular yield inherent to non-invasive CIC sampling in severe dry eye conditions, the recovered strips were utilized exclusively for total RNA extraction to ensure uncompromised transcriptomic fidelity, precluding parallel protein or immunofluorescence assays.

### 2.3. RNA Sequencing and Data Download

Following total RNA extraction, RNA sequencing was performed on the Illumina platform. The obtained raw reads underwent quality control and were then aligned to the human reference genome (hg19) using the STAR software (version 2.7.10a) to determine the genomic mapping of the sequencing reads and generate a gene expression matrix. Subsequently, gene-level counting was performed using featureCounts under default parameters. This self-generated sequencing dataset served as the discovery cohort and has been deposited in the NCBI SRA repository under the BioProject accession number PRJNA1348748. An independent public dataset, GSE176510, was sourced from the GEO database (platform GPL28577). This dataset comprises 7 SSDE samples and 19 healthy control samples and serves as an independent external validation cohort to evaluate the expression trends of the candidate target genes. The two datasets originated independently and were not merged at the raw sample level, ensuring complete methodological independence between the discovery and validation phases.

### 2.4. Identification of Differentially Expressed Genes and OSRGs

Identification of DEGs: Differential expression analysis between the SSDE group and the healthy control group was exclusively performed using the limma package in R, utilizing the log2-transformed FPKM expression matrix as input. To capture a sufficiently broad transcriptomic landscape for downstream functional enrichment analyses, we applied an exploratory screening threshold of |log2FC| > 0 and a nominal (unadjusted) *p*-value < 0.05 (as no individual genes met the stringent threshold of adjusted *p* < 0.05 after Benjamini–Hochberg correction). Consequently, the resultant 1826 differentially expressed genes (DEGs) (Table S2) are strictly defined as exploratory candidate DEGs. Volcano plots and heatmaps were generated utilizing the ggplot2 and pheatmap packages to visualize these expression patterns.

Identification of OSRGs and OSRDEGs: To construct a comprehensive candidate set of oxidative stress-related genes (OSRGs), we integrated database retrieval with curated datasets. Initially, the GeneCards [14] database (https://www.genecards.org/) was queried using the keyword “oxidative stress” (accessed: 27 September 2024). Protein-coding genes with a relevance score > 0 were retained, establishing a foundational set of 1780 candidate OSRGs. To further expand and cross-validate this repertoire, we incorporated three functionally validated OSRG lists derived from previously published transcriptomic studies [15,16,17] (comprising 279, 60, and 467 genes, respectively (Table S3)).

Merging these three curated lists with the foundational 1780 GeneCards genes established a comprehensive master set of 2411 candidate OSRGs after deduplication. Next, this master set was intersected with the external validation cohort. Because the GSE176510 dataset utilized a targeted NanoString immune gene panel containing a limited background of 579 genes, exactly 244 OSRGs were successfully mapped to this expression matrix. Finally, these 244 mapped genes were intersected with our 1826 exploratory candidate DEGs, yielding 12 core OSRDEGs (oxidative stress-related DEGs) for subsequent evaluation in the external cohort. This intersection was visualized using a Venn diagram.

### 2.5. Gene Ontology (GO) and Pathway (KEGG) Enrichment Analysis

To comprehensively elucidate the global transcriptomic alterations and pathway dysregulations induced by the disease microenvironment, enrichment analyses of Gene Ontology (GO) [18] terms and Kyoto Encyclopedia of Genes and Genomes (KEGG) [19] pathways were performed on the entire list of 1826 exploratory candidate DEGs using the clusterProfiler R package (v4.10.0) [20]. Significantly enriched terms were defined by a *p*-value < 0.05 and a false discovery rate (FDR) < 0.25, with *p*-values adjusted by the Benjamini–Hochberg method.

### 2.6. Gene Set Enrichment Analysis (GSEA)

Gene Set Enrichment Analysis (GSEA) [21] was performed on the entire ranked list of expressed genes derived from the SSDE dataset using the clusterProfiler R package10 (v4.10.0) with 1000 permutations and a fixed seed of 2020. Analysis included gene sets ranging from 10 to 500 genes, sourced from the Molecular Signatures Database (MSigDB) [22] c2.cp.all.v2022.1.Hs.symbols.gmt file, encompassing 3050 canonical pathways. Significant enrichment was defined by *p* < 0.05 and FDR < 0.25, with *p*-values adjusted by the Benjamini–Hochberg method.

### 2.7. Protein–Protein Interaction (PPI) Network and Hub Gene Selection

This research utilized the STRING database [23] to construct a PPI network founded on OSRDEGs, setting a threshold for the minimum interaction score at 0.15 (indicative of low confidence). Genes exhibiting interactions with other entities within the PPI network were earmarked for subsequent examination. We utilized Cytoscape (version 3.9.1) [24] software along with the CytoHubba [25] plugin, implementing five distinct algorithms: Maximal Clique Centrality (MCC), Radiality, Degree, BottleNeck, and Stress. Within the PPI framework, scores for the OSRDEGs were calculated, resulting in the identification of the top 10 candidate hub genes based on these scores. Oxidative stress-related candidate hub genes were identified by intersecting candidate genes from five algorithms using a Venn diagram. In addition, we harnessed the GeneMANIA [26] online resource to explore functional interaction networks associated with the hub genes, thereby augmenting our systemic biological interpretation.

### 2.8. Regulatory Network Construction

Transcription factors (TFs) regulating the candidate hub genes were identified using the ChIPBase database [27] (http://rna.sysu.edu.cn/chipbase/, accessed on 20 October 2024). The influence of these transcription factors on hub genes was investigated, and the resulting mRNA–TF regulatory network was visualized using Cytoscape. Additionally, we predicted potential RNA-binding proteins (RBPs) [28] that might target the candidate hub genes by utilizing the StarBase v3.0 database (https://starbase.sysu.edu.cn/). The mRNA-RBP regulatory network was also constructed and represented visually through Cytoscape.

### 2.9. Correlation Analysis and Diagnostic ROC Evaluation

To compare SSDE patients with control samples, Spearman correlation analysis was performed to assess the inter-regulatory relationships among the candidate biomarkers’ hub genes based on their expression in both the SSDE and GEO datasets. The results were visually represented through correlation heatmaps and scatter plots. We categorized the correlation coefficients (r values) into several groups: weak or negligible correlation for |r| < 0.3, weak correlation for 0.3 ≤ |r| < 0.5, moderate correlation for 0.5 ≤ |r| < 0.8, and strong correlation for |r| ≥ 0.8. Furthermore, we employed the R package pROC [29] (Version 1.18.5) to create receiver operating characteristic (ROC) curves for the candidate biomarkers and to compute the area under the curve (AUC) values. This analysis was employed to assess the exploratory discriminative potential of these transcriptomic signatures between the SSDE and healthy cohorts. Area under the curve (AUC) values range from 0.5 to 1.0, with values closer to 1.0 indicating higher statistical performance in our dataset.

### 2.10. Immune Infiltration Analysis of SSDE Dataset (CIBERSORT)

To explore the preliminary immune microenvironment landscape associated with oxidative stress in SSDE, the CIBERSORT [30] algorithm, along with the LM22 signature gene matrix, was employed to estimate the proportions of 22 immune cell types within the SSDE dataset. Immune cell fractions with estimated enrichment scores greater than zero were retained for subsequent analyses, revealing exploratory infiltration patterns that were visualized in a bar chart. Following this, we computed the correlations among the various immune cells using the Spearman method and visualized these correlations through a heatmap, generated with the R package pheatmap (Version 1.0.12). Correlations between the candidate biomarkers and immune cells were assessed to explore the complex interplay between oxidative stress and localized immune responses, retaining associations with a nominal *p* < 0.05. Given the small cohort size and the reliance on uncorrected correlation testing, these immune infiltration findings are strictly defined as exploratory. Results were visualized using a correlation bubble plot generated with ggplot2 (v3.4.4).

### 2.11. Real-Time PCR

Total RNA was extracted from conjunctival samples using TRIzol reagent (Invitrogen, Carlsbad, CA, USA), with modifications to accommodate the cellulose acetate membrane. This rigorous RNA extraction procedure fully consumed the limited clinical specimens. RNA purity was assessed by A260/A280 (1.8–2.2) and A260/A230 (>2.0) ratios. One microgram of total RNA was subjected to genomic DNA removal followed by reverse transcription using an Evo M-MLV RT Kit (Accurate Biotechnology, Changsha, China) to synthesize cDNA. The primer sequences for the selected disease-associated candidate biomarkers (*CDKN1A*, *BAX*, *TGFBR1*, *S100A9*) and the housekeeping gene *GAPDH* are listed in Table 1. Each 10 μL qRT-PCR reaction contained 5 μL of SYBR Green Master Mix (2×, Low ROX) (Accurate Biotechnology, Changsha, China), 0.4 μM of each primer, 1 μL of cDNA, and ddH_2_O. The amplification program was: 95 °C for 5 min, followed by 40 cycles of 95 °C for 10 s and 60 °C for 30 s, with a final melting curve step. All reactions were performed in triplicate, and relative gene expression levels were calculated using the 2^−ΔΔ*C_T_*^ method.

### 2.12. Animal Models and Ethical Approval

Twelve-week-old female non-obese diabetic (NOD) mice and age-matched BALB/c mice were utilized in this study. The animals were housed under standard specific pathogen-free (SPF) conditions with a 12 h light/dark cycle and provided ad libitum access to standard laboratory chow and water. All animal care and experimental procedures were performed in strict accordance with the Guidelines for the Care and Use of Laboratory Animals. The study protocol was reviewed and formally approved by the Institutional Animal Care and Use Committee (IACUC) of Chongqing Medical University (Approval No. IACUC-CQMU-2024-0855).

### 2.13. Measurement of Lipid Peroxidation (MDA Assay)

To provide supportive cross-species and cross-tissue biological context for the local oxidative stress status, the levels of malondialdehyde (MDA), a well-established end-product of lipid peroxidation, were quantified in the extraorbital lacrimal glands of 12-week-old NOD mice (*n* = 6) and age-matched BALB/c controls (*n* = 6). Briefly, the freshly harvested bilateral extraorbital lacrimal glands were carefully isolated from surrounding connective tissues and precisely weighed. The tissues were immediately homogenized in ice-cold phosphate-buffered saline (PBS) at a strict weight-to-volume ratio of 1:9 to yield 10% (*w/v*) homogenates. After centrifugation at 10,000× *g* for 10 min at 4 °C, the supernatants were collected. MDA concentrations were determined using a Lipid Peroxidation MDA Assay Kit (Beyotime Biotechnology, Haimen, China) based on the thiobarbituric acid (TBA) colorimetric method, according to the manufacturer’s instructions. The absorbance was measured at 532 nm using a microplate reader. After subtracting the blank background absorbance, sample concentrations were calculated against a standard curve. To account for potential disease-related glandular atrophy, the final MDA levels were rigorously normalized to the wet tissue weight and expressed as nmol/mg tissue.

### 2.14. Statistical Analysis

All data processing and analyses were performed using R software (v4.2.2). The normality of the data distribution was assessed using the Shapiro–Wilk test, and the homogeneity of variances was evaluated using Levene’s test. Comparisons between two groups were conducted using the standard Student’s *t*-test for normally distributed data with equal variances. When data were normally distributed but exhibited unequal variances, Welch’s *t*-test was applied. For non-normally distributed data, the non-parametric Mann–Whitney U test was utilized. Two-tailed *p*-values < 0.05 were considered statistically significant across all evaluations unless otherwise specified.

### 2.15. Molecular Docking and Molecular Dynamics Simulations

To computationally explore potential pharmacological interventions, AutoDock Vina 1.2.3 [31] was employed for molecular docking of the natural antioxidants (ursolic acid and andrographolide) with the selected disease-associated candidate molecular nodes (CDKN1A and BAX). Subsequently, 100 ns all-atom molecular dynamics (MD) simulations were performed using AMBER 24 under the ff14SB [32] and GAFF2 [33] force fields. The theoretical structural stability of the in silico complexes was assessed using Root Mean Square Deviation (RMSD), radius of gyration (Rg), and hydrogen bonds. The binding free energy was computationally estimated using the MM/GBSA method.

## 3. Results

### 3.1. Patient Demographics and Clinical Characteristics

A total of 31 female participants were enrolled in this study, comprising 16 patients with SSDE and 15 healthy controls. Age distribution showed no statistical difference between the two groups. To comprehensively assess ocular surface damage and tear film dysfunction, key clinical parameters were evaluated, including the Ocular Surface Disease Index (OSDI), tear film break-up time (TBUT), Schirmer I test (SIT), and corneal fluorescein staining (CFS). As detailed in Table 2, the SSDE group exhibited severe ocular surface deterioration compared to healthy controls, characterized by a markedly elevated OSDI score, significantly reduced TBUT and SIT values, and extensive punctate corneal epithelial staining.

To explore the underlying molecular changes, a representative subgroup comprising 9 SSDE patients and 7 healthy controls underwent RNA sequencing, with their detailed demographic and specific clinical information listed in Table 3. Conjunctival samples from all 31 participants were utilized for the RT-qPCR experiments.

### 3.2. Oxidative Stress-Related Differentially Expressed Genes (OSRDEGs) in SSDE Dataset

**Figure 1:**
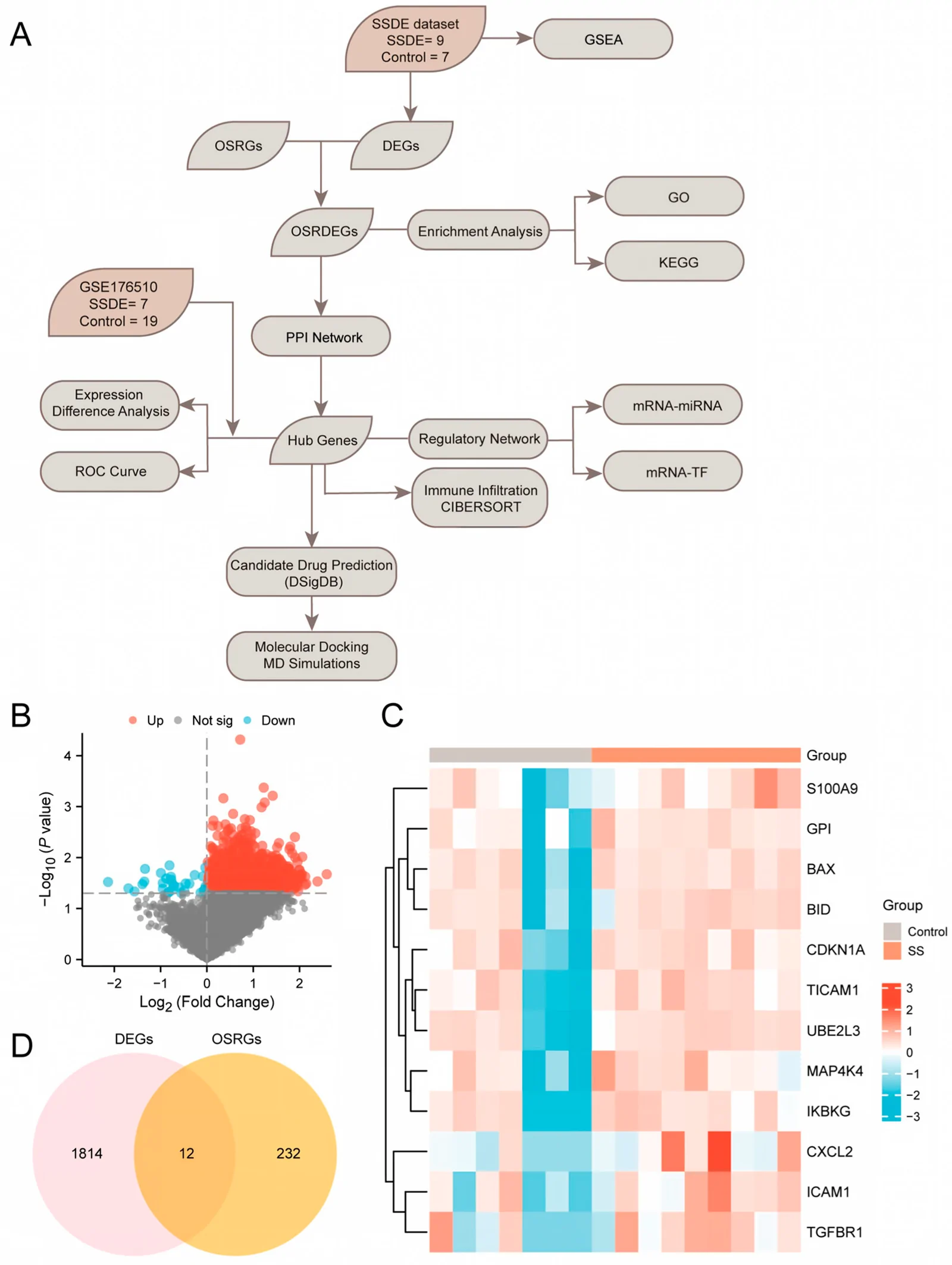
Differential gene expression analysis. Study workflow and OSRDEG analysis. (**A**) Flowchart of this study. (**B**) Volcano plot of the 1826 exploratory candidate DEGs in SSDE. (**C**) Heatmap of the 12 OSRDEG expression in SSDE. (**D**) Venn diagram of DEGs and OSRGs showing the intersection of 12 core genes. GSEA: Gene Set Enrichment Analysis; DEGs: differentially expressed genes; OSRGs: oxidative stress-related genes; GO: Gene Ontology; KEGG: Kyoto Encyclopedia of Genes and Genomes; OSRDEGs: oxidative stress-related differentially expressed genes; PPI: protein–protein interaction network; ROC: receiver operating characteristic; TF: transcription factor.

The workflow for this study is illustrated in Figure 1A. Following the aforementioned methods, we identified 1826 exploratory candidate DEGs from the SSDE dataset, comprising 1794 upregulated genes and 32 downregulated genes, reflecting the global transcriptomic alterations in the diseased ocular surface. A volcano plot was generated to visualize these global DEG results (Figure 1B). To explore the potential oxidative stress-mediated transcriptomic signature, we intersected the 1826 DEGs with the 244 tissue-expressed OSRGs and visualized the results using a Venn diagram (Figure 1D). This analysis yielded a core set of 12 candidate OSRDEGs: *TICAM1*, *CXCL2*, *ICAM1*, *MAP4K4*, *UBE2L3*, *BAX*, *IKBKG*, *GPI*, *BID*, *CDKN1A*, *TGFBR1*, and *S100A9*. Based on the intersection, we evaluated expression changes in these 12 OSRDEGs across sample groups in the SSDE dataset, visualized by a heatmap (Figure 1C).

### 3.3. Gene Ontology (GO) and Pathway (KEGG) Enrichment Analysis

**Figure 2:**
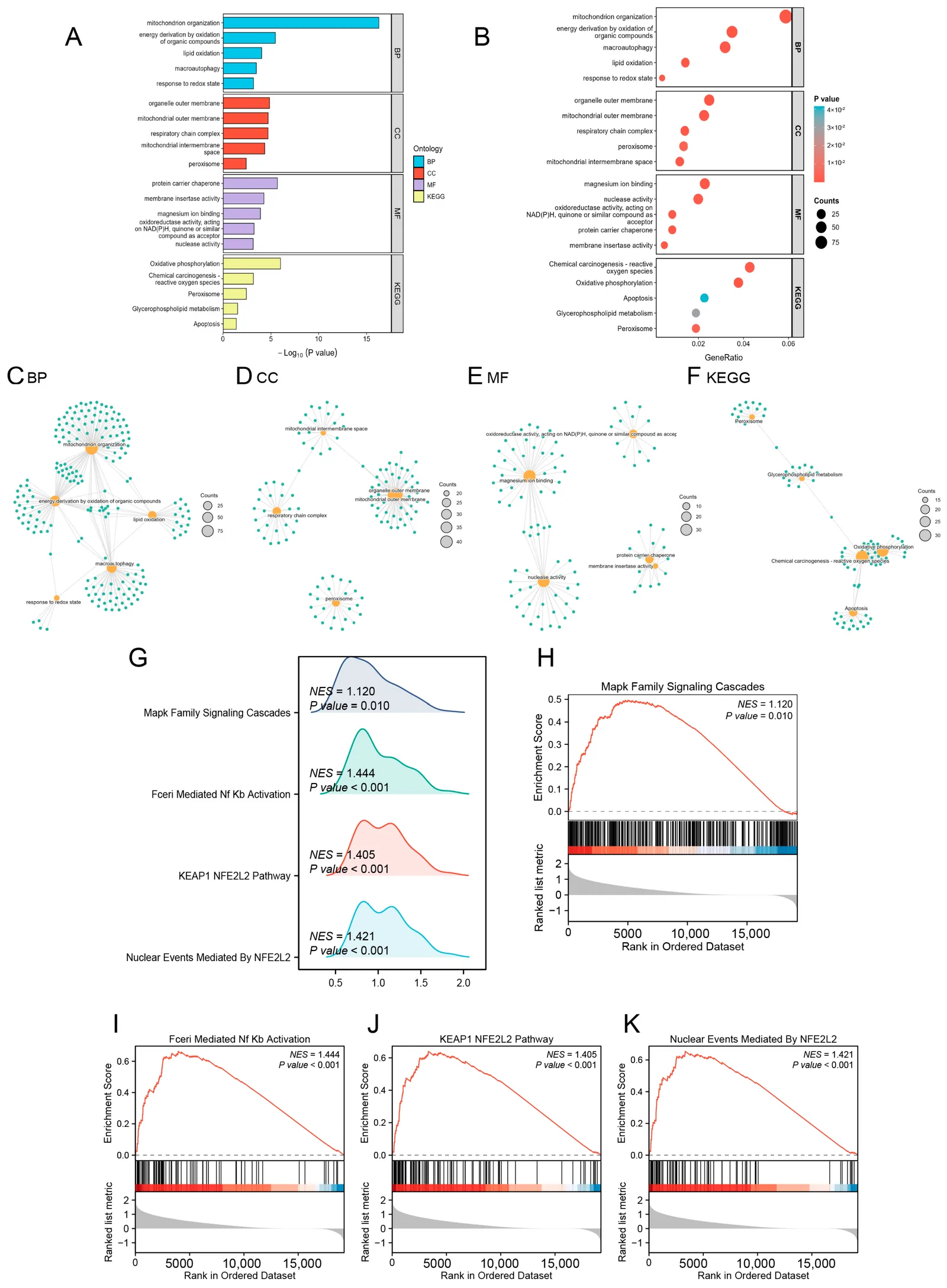
Pathway enrichment and gene set enrichment analysis. (**A**) Bar plot. (**B**) Bubble plot. (**C**–**F**) Network diagrams for biological processes (BP), cellular components (CC), molecular functions (MF), and KEGG pathways. (**G**–**K**) Mountain plot of enriched biological functions. (**H**–**K**) Enrichment in MAPK signaling (**H**), FcεRI-NF-κB activation (**I**), KEAP1-NFE2L2 (**J**), and NFE2L2 nuclear events (**K**). The horizontal dashed line represents the baseline where the enrichment score is zero. The red-to-blue color gradient represents the ranked gene list, with red indicating a positive correlation (upregulation) and blue indicating a negative correlation (downregulation).

To comprehensively evaluate the biological implications of the transcriptomic alterations in the SSDE dataset, GO and KEGG enrichment analyses were performed on the entire global list of 1826 candidate DEGs. This unbiased global landscape revealed an exploratory transcriptomic signature dominated by oxidative stress, lipid damage, and programmed cell death (Figure 2). In the Biological Process (BP) category, the DEGs were significantly enriched in processes reflecting a severe stress microenvironment, specifically “response to redox state,” “lipid oxidation,” and “macroautophagy.” These processes potentially underscore the occurrence of severe lipid peroxidation and the subsequent activation of cellular clearance and inflammatory-like mechanisms in response to extreme oxidative damage. Furthermore, pathways such as “mitochondrion organization” and “energy derivation by oxidation of organic compounds” suggest a potential disruption of cellular energetic homeostasis. For cellular components (CC), the altered genes were predominantly localized to the “peroxisome”—the primary organelle responsible for reactive oxygen species (ROS) scavenging—and critical mitochondrial compartments, notably the “mitochondrial outer membrane,” “mitochondrial intermembrane space,” and “respiratory chain complex.” The pronounced involvement of the mitochondrial outer membrane is highly consistent with the classical mechanisms of mitochondria-dependent apoptosis, serving as the primary site where pro-apoptotic proteins disrupt membrane permeability. Molecular function (MF) analysis further supported this stress-induced apoptotic landscape. Significant enrichment in “oxidoreductase activity (acting on NAD(P)H, quinone or similar compound as acceptor)” indicated an active, yet overwhelmed, cellular struggle to maintain redox balance. Additionally, the enrichment of “nuclease activity,” “protein carrier chaperone,” and “membrane insertase activity” strongly correlates with the execution phase of apoptosis (such as DNA fragmentation) and the cellular attempt to manage misfolded proteins and membrane damage under extreme oxidative stress.

Crucially, KEGG pathway analysis provided exploratory bioinformatic indications of the oxidative stress and apoptotic cascade. The DEGs were prominently enriched in the “Chemical carcinogenesis—reactive oxygen species” and “Peroxisome” pathways, suggesting a potential link to ROS accumulation in SSDE. The significant enrichment of “Apoptosis” perfectly aligns with the extensive cellular death observed on the ocular surface. Moreover, the presence of “Glycerophospholipid metabolism” and “Oxidative phosphorylation” further supports the bioinformatic hypothesis of disrupted cell membrane integrity (lipid peroxidation) and mitochondrial energetic collapse. Collectively, these results, visually depicted in the enrichment bubble plots (Figure 2A,B), highlight an exploratory molecular association from local oxidative stress and lipid peroxidation to terminal apoptosis in the conjunctival epithelium of SSDE patients.

Figure 2’s GO and KEGG enrichment results were visualized using bar and bubble plots (Figure 2A,B). Network diagrams for the BP, CC, MF, and KEGG pathways were generated from the enrichment analysis (Figure 2C–F). Node size reflects the number of associated molecules, illustrating the relationships between genes and their functional annotations.

### 3.4. Gene Set Enrichment Analysis (GSEA)

To complement the global DEG enrichment analysis and provide an unbiased, threshold-free evaluation of transcriptomic alterations, GSEA was performed on the entire transcriptome profile of the SSDE dataset to assess associations between gene expression and biological processes, cellular components, and molecular functions (Figure 2G). The analysis revealed significant enrichment of genes in the SSDE dataset for pathways related to oxidative stress, inflammation, and cell survival. Notable biological functions and signaling pathways included the Mapk Family Signaling Cascades (Figure 2H), FcεRI Mediated NF-κB Activation (Figure 2I), KEAP1-NFE2L2 Pathway (Figure 2J), and Nuclear Events Mediated by NFE2L2 (Figure 2K). Crucially, the prominent activation of the KEAP1-NFE2L2 axis—the master transcriptional regulator of cellular antioxidant responses—alongside NF-κB and MAPK inflammatory cascades, provided an exploratory, data-driven rationale for our specific focus on oxidative stress-mediated networks in the subsequent analyses.

### 3.5. Elevated Lipid Peroxidation Provides Supportive Evidence for the Oxidative Stress Microenvironment In Vivo

Driven by the prominent enrichment of “lipid oxidation” and cell death pathways (such as “ferroptosis” and “apoptosis”) in our global DEG analysis, we sought to biochemically explore the presence of oxidative stress in vivo. We measured the levels of MDA, a classic hallmark of lipid peroxidation and oxidative damage, in the lacrimal glands of SSDE mice.

**Figure 3:**
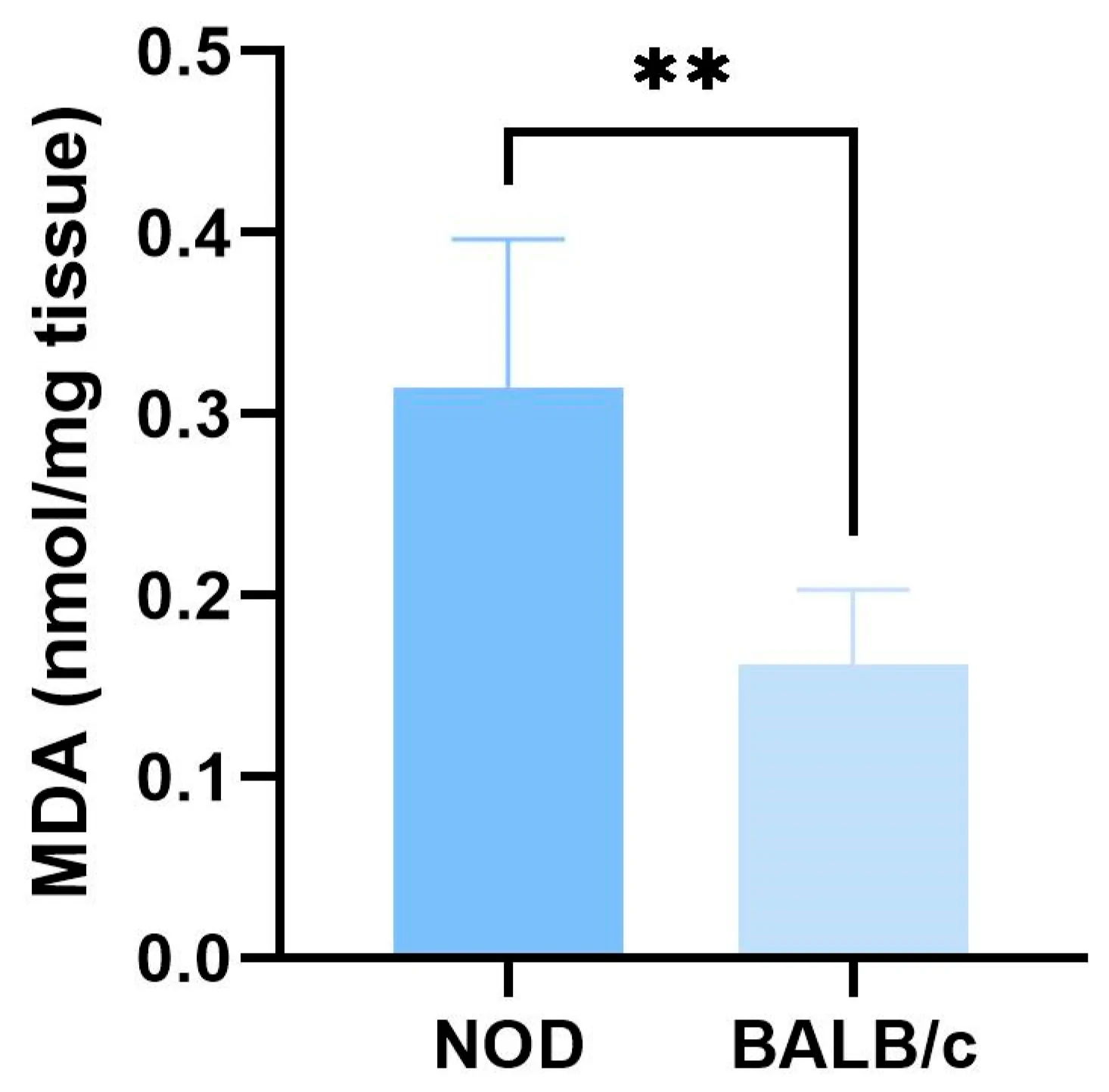
Elevated lipid peroxidation in the lacrimal glands of SSDE mice in vivo. Malondialdehyde (MDA) concentration (nmol/mg tissue) was quantified as a biochemical hallmark of oxidative stress in the lacrimal glands of NOD mice (SSDE model) and age-matched wild-type BALB/c controls. The MDA levels were significantly elevated (approximately 1.8-fold) in the NOD group compared to the healthy controls, providing supportive biological context for the bioinformatic prediction of the oxidative stress microenvironment. Data are presented as mean ± SD (n = 6 per group). Statistical significance was determined using an unpaired Student’s *t*-test. ** p < 0.01 versus BALB/c controls.

Consistent with our exploratory transcriptomic predictions, the biochemical assay revealed a profound accumulation of lipid peroxides in the disease model. The MDA levels in the lacrimal glands of NOD mice were significantly elevated by approximately 1.8-fold compared to those in the healthy wild-type BALB/c controls (0.315 ± 0.08 vs. 0.175 ± 0.04 nmol/mg tissue, *p* < 0.01) (Figure 3). While representing cross-species and cross-tissue data, this in vivo assay provides supportive, albeit indirect, biological context for our bioinformatic findings, suggesting that severe local oxidative stress and unabated lipid peroxidation are fundamental pathological features characterizing the SSDE microenvironment.

### 3.6. Protein–Protein Interaction (PPI) Network and Candidate Hub Gene Selection

**Figure 4:**
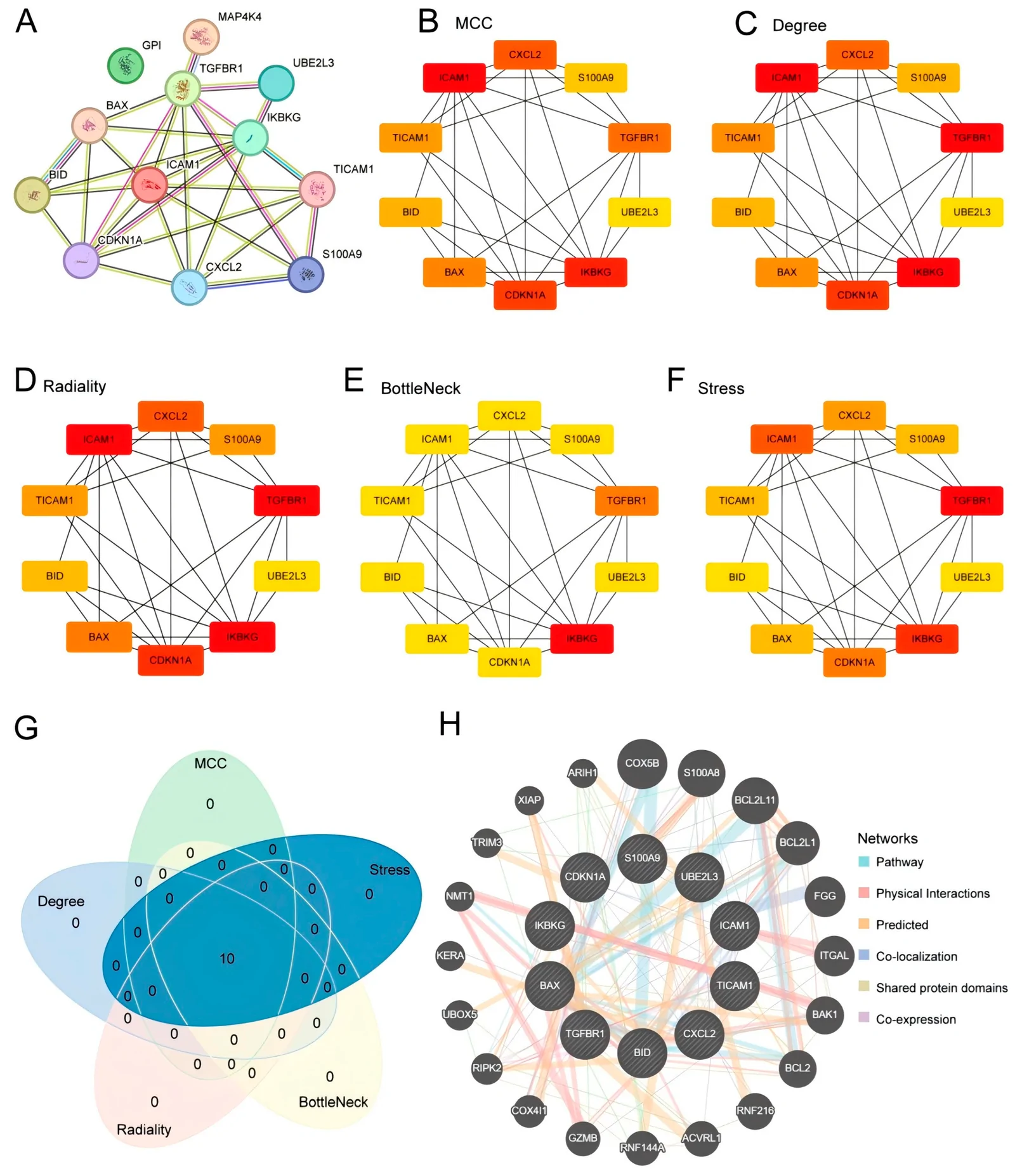
PPI network and hub gene analysis. (**A**) STRING-derived PPI network. (**B**–**F**) Top 10 OSRDEGs ranked by MCC (**B**), Degree (**C**), Radiality (**D**), BottleNeck (**E**), and Stress (**F**). (**G**) Venn diagram of overlapping hub genes. (**H**) GeneMANIA interaction network. In subfigures (B–F), the node color gradient from yellow to red represents the topological score of each hub gene calculated by the respective cytoHubba algorithms, with darker red indicating a higher score (greater essentiality).

Using the STRING database, we constructed a PPI network encompassing the 12 core OSRDEGs (Figure 4A) to evaluate their topological significance. The results indicated that 11 OSRDEGs were interconnected, namely: *TICAM1*, *CXCL2*, *ICAM1*, *MAP4K4*, *UBE2L3*, *BAX*, *IKBKG*, *BID*, *CDKN1A*, *TGFBR1*, and *S100A9*. Subsequently, we utilized the CytoHubba plugin in Cytoscape to compute scores for these 11 OSRDEGs using five algorithms: Maximal Clique Centrality (MCC), Radiality, Degree, BottleNeck, and Stress. Based on the scores, we prioritized the OSRDEGs and displayed the top 10 genes from each method: MCC (Figure 4B), Degree (Figure 4C), Radiality (Figure 4D), BottleNeck (Figure 4E), and Stress (Figure 4F). In these visualizations, node colors transition from red to yellow, representing high-to-low scores, respectively. Subsequently, we conducted an intersection analysis of the top-ranked genes identified by five independent algorithms, with the overlapping results depicted in a Venn diagram (Figure 4G). The intersecting genes were designated as candidate hub genes associated with the SSDE dataset, comprising 10 candidate hub core genes: *CDKN1A*, *BAX*, *TICAM1*, *ICAM1*, *BID*, *TGFBR1*, *UBE2L3*, *IKBKG*, *CXCL2*, and *S100A9*. Additionally, we employed the GeneMANIA platform to predict an interaction network comprising the 10 candidate hub genes and their functionally related counterparts (Figure 4H). The diverse colored edges denote co-expression, shared protein domains, and additional interaction types.

### 3.7. Construction of Upstream Regulatory Networks

**Figure 5:**
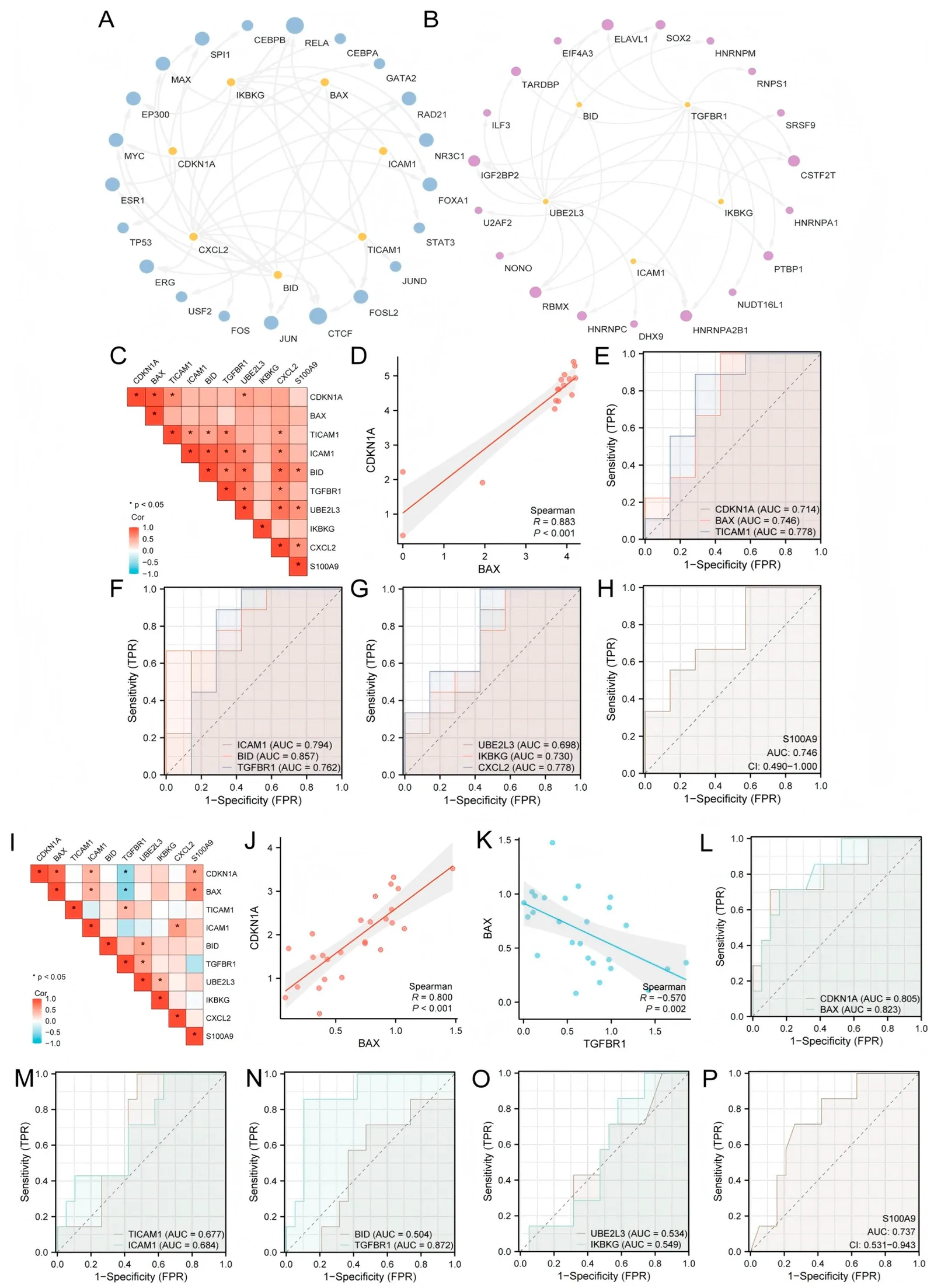
Construction of regulatory networks and exploratory candidate biomarker correlations and ROC analysis. (**A**) mRNA-TF network (orange: mRNA; blue: TFs). (**B**) mRNA-RBP network (orange: mRNA; purple: RBPs). (**C**) Correlation heatmap in SSDE database. (**D**) CDKN1A-BAX scatter plot. (**E**–**H**) ROC curves for hub genes. (**I**) Correlation heatmap in GSE176510. (**J**,**K**) Scatter plots for top correlations. (**L**–**P**) ROC curves for hub genes in GSE176510. In subfigures (**E**–**H**) and (**L**–**P**), the diagonal dashed line represents the reference line for a random classifier (AUC = 0.5).

First, to explore the upstream transcriptional regulatory mechanisms, we retrieved transcription factors (TFs) associated with the candidate hub genes using the ChIPBase database to construct the mRNA-TF regulatory network. This network was visualized using Cytoscape (Figure 5A) and includes 7 candidate hub genes and 21 transcription factors. Next, to investigate potential post-transcriptional regulation, we predicted RNA-binding proteins (RBPs) related to the candidate hub genes through the StarBase database, resulting in the creation of the mRNA-RBP regulatory network, which was visualized with Cytoscape (Figure 5B). This network comprises 5 candidate hub genes and 19 RNA-binding proteins.

### 3.8. Correlation Analysis and ROC Curve Analysis of Candidate Hub Genes in the SSDE Dataset

We performed a correlation analysis using the full expression matrix of the 10 candidate biomarkers within the SSDE dataset and displayed the findings with a correlation heatmap (Figure 5C). The analysis indicated a generally positive correlation among the genes. Subsequently, we illustrated the correlation results of the top positively correlated genes using a scatter plot (Figure 5D), revealing a strong positive correlation between CDKN1A and BAX (*p*-value < 0.05, r value = 0.883), two candidate molecular nodes commonly associated with cellular stress and apoptosis.

We employed the R package pROC to generate receiver operating characteristic (ROC) curves based on the expression profiles of the candidate biomarkers in the SSDE dataset. In the comparison between SSDE and control samples, the expression levels of CDKN1A, BAX, TICAM1, ICAM1, BID, TGFBR1, IKBKG, CXCL2, and S100A9 as shown by the resulting curves (Figure 5E–H) demonstrated moderate discriminative power, with area under the curve (AUC) values ranging from 0.7 to 0.9. By contrast, UBE2L3 showed a lower classification performance (AUC between 0.5 and 0.7). These results suggest that nine of the ten candidates possess appreciable exploratory discriminative potential between the SSDE and healthy cohorts.

### 3.9. External Validation of Correlation Analysis and Exploratory ROC Curve Analysis of Candidate Hub Genes in GSE176510

Following the exclusion of the gene with a low AUC, we performed a correlation analysis using the full expression matrix of the remaining nine candidate biomarkers from the GSE176510 dataset. The results were visualized as a correlation heatmap (Figure 5I), indicating a generally positive correlation among the genes. We then illustrated the correlation results for the top positively and negatively correlated genes using scatter plots (Figure 5J,K). The study found a strong positive correlation between CDKN1A and BAX in GSE176510 (*p*-value < 0.05, r value = 0.800), consistent with our discovery dataset, and a significant negative correlation between BAX and TGFBR1 (*p*-value < 0.05, r value = −0.570).

We employed the R package pROC to generate ROC curves based on the expression levels of the candidate biomarkers in the GSE176510 dataset. The resulting curves (Figure 5L–P) showed that CDKN1A, BAX, TGFBR1, and S100A9 demonstrated moderate discriminatory potential (AUC between 0.7 and 0.9) in distinguishing SS samples from controls. Conversely, the expression levels of TICAM1, ICAM1, BID, and UBE2L3 exhibited lower classification accuracy (0.5 < AUC < 0.7). These results suggest that the four hub genes might serve as potential exploratory biomarkers for SSDE, reflecting an exploratory oxidative stress-mediated apoptotic and inflammatory signature across independent cohorts.

### 3.10. Immune Infiltration Analysis of SSDE (CIBERSORT)

**Figure 6:**
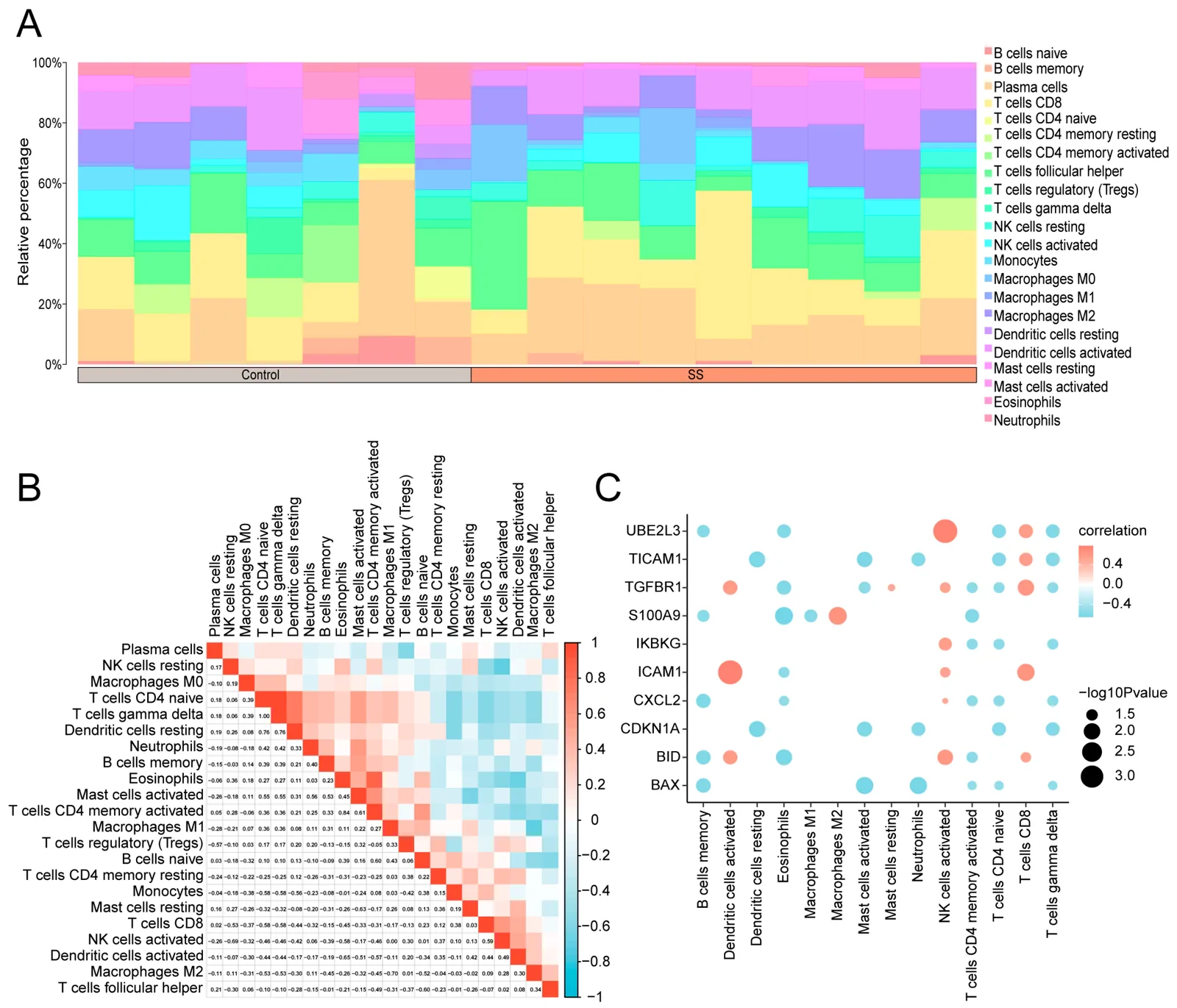
Immune infiltration analysis in SSDE. (**A**) Immune cell proportions. (**B**) Immune cell correlation heatmap. (**C**) Hub gene-immune cell correlations. Correlation strength: |r| < 0.3 (weak/none), 0.3–0.5 (weak), 0.5–0.8 (moderate), >0.8 (strong). SSDE samples: light orange; controls: light gray.

We applied the CIBERSORT algorithm to calculate the proportions of 22 immune cell types in the SSDE dataset. Following this, Spearman correlation analysis was performed to assess the exploratory relationships among these immune cell populations. The results, visualized in a bar plot, revealed the relative proportions of immune cell subsets across the samples (Figure 6A). Subsequently, we visualized the correlation of immune cell infiltration abundances with a heatmap (Figure 6B). The study showed a significant positive relationship between the abundance levels of activated CD4+ memory T cells and eosinophils (r value = 0.843). In contrast, the abundance levels of M1/M2 macrophages exhibited a strong negative correlation (r value = −0.704). In conclusion, the associations between candidate biomarker expression and immune cell infiltration were illustrated using a bubble chart (Figure 6C). Notably, ICAM1 exhibited a strong positive correlation with activated dendritic cells (r = 0.775, *p* < 0.05), whereas S100A9 showed a significant negative correlation with eosinophils (r = –0.651, *p* < 0.05). Given the lack of formal correction for multiple testing in these correlation analyses, these findings provide preliminary computational indications highlighting a potential link between the identified oxidative stress signature and the dysregulated immune microenvironment characteristic of SSDE.

### 3.11. Transcriptomic Evaluation of Candidate Biomarkers

**Figure 7:**
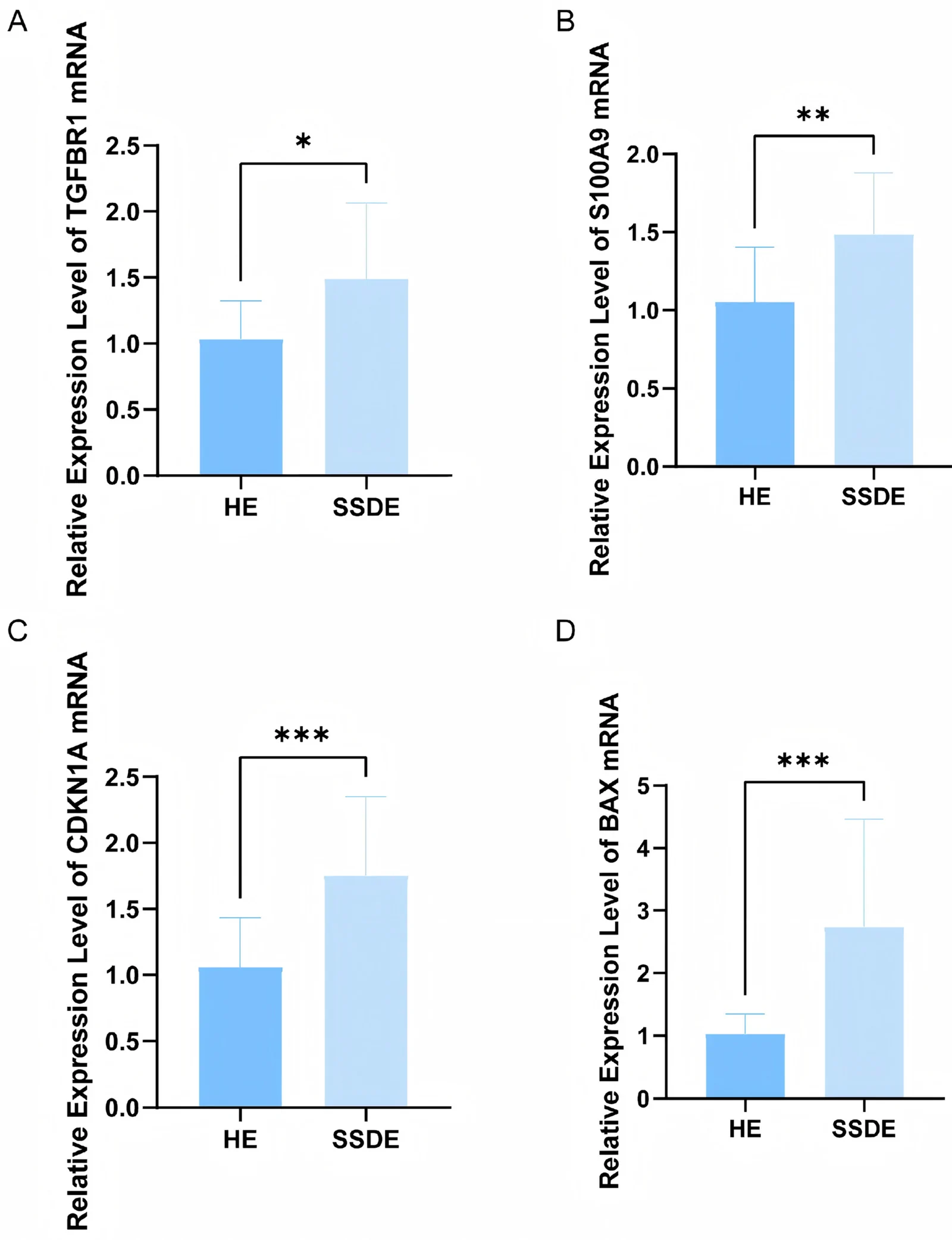
The expression levels of *CDKN1A*, *BAX*, *TGFBR1*, and *S100A9*. (**A**) The expression levels of *TGFBR1*. (**B**) The expression levels of *BAX*. (**C**) The expression levels of *S100A9*. (**D**) The expression levels of *CDKN1A*. * *p* < 0.05, ** *p* < 0.01, *** *p* < 0.001.

The genes *CDKN1A*, *BAX*, *TGFBR1*, and *S100A9* were identified as core disease-associated candidate biomarkers through transcriptome sequencing and bioinformatics analysis. To experimentally evaluate these findings at the transcriptional level, conjunctival epithelial specimens were collected from the aforementioned clinical cohorts using CIC, and quantitative RT-qPCR was performed. As rigorously detailed in our methodology, the extremely limited cellular yield from non-invasive CIC sampling was fully utilized to ensure the high sensitivity and reliability of this quantitative RNA assay. As shown in Figure 7, the expression levels of *CDKN1A, BAX, TGFBR1*, and *S100A9* were significantly upregulated in SSDE patients compared with healthy controls (* *p* < 0.05, ** *p* < 0.01, *** *p* < 0.001) (Figure 7A–D), which was consistent with the exploratory results of the computational transcriptome sequencing.

### 3.12. In Silico Prediction of Pharmacological Candidates

**Figure 8:**
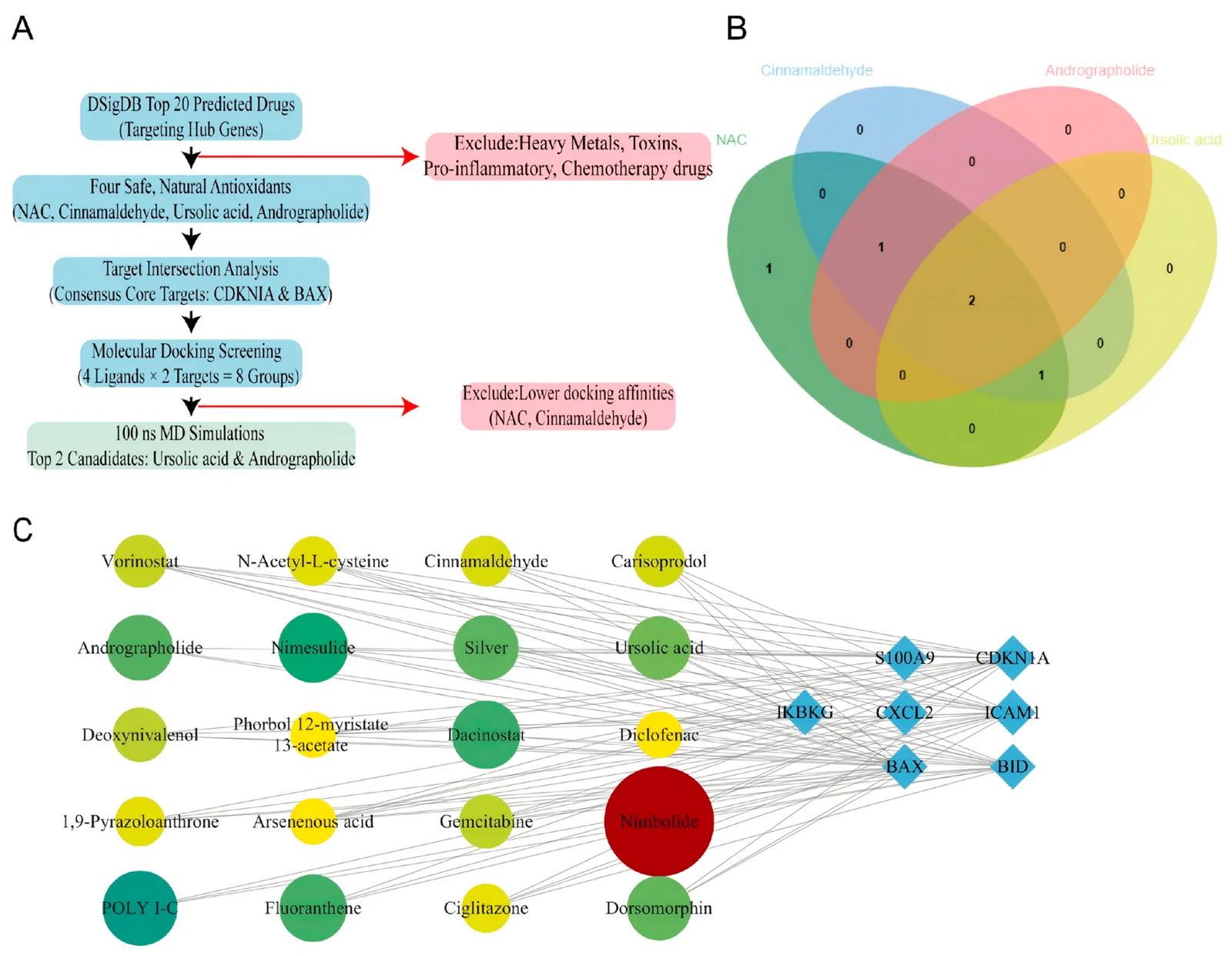
Candidate drug prediction, hierarchical screening, and molecular node identification for SSDE. (**A**) The hierarchical filtering flowchart of the top 20 candidate drugs predicted by the DSigDB database. Heavy metals, pro-inflammatory agents, and cytotoxic chemotherapy drugs were sequentially excluded to isolate four natural antioxidants (N-acetyl-L-cysteine, cinnamaldehyde, andrographolide, and ursolic acid). (**B**) The drug–target interaction network of the four screened natural antioxidants and their corresponding predicted candidate targets, the numbers within the Venn diagram represent the count of unique and overlapping candidate targets among the four screened natural antioxidants. (**C**) Venn diagram illustrating the target intersection of the four candidate drugs, revealing that CDKN1A and BAX are the exclusively shared consensus molecular nodes within this computational framework.

We computationally explored potential interacting candidates associated with the evaluated candidate biomarkers using the Drug Signatures Database (DSigDB) accessed through the Enrichr platform. To ensure high clinical translational value, the initial top 20 predicted drugs underwent a strict hierarchical filtering process (Figure 8A and Table S4). By excluding heavy metals, pro-inflammatory agents, and cytotoxic chemotherapy drugs, four natural antioxidant compounds—N-Acetyl-L-cysteine, cinnamaldehyde, andrographolide, and ursolic acid—emerged as the primary candidates for structural exploration.

To determine the precise molecular nodes for structural evaluation, we constructed a drug–target interaction network (Figure 8B) and performed intersection analysis (Figure 8C). Interestingly, the targeting profiles of all four candidates converged specifically on CDKN1A and BAX, indicating that these two genes serve as the consensus molecular nodes within this predictive network. Subsequently, we obtained the 3D structures of CDKN1A, BAX, and the four ligands to perform initial molecular docking using AutoDock Vina 1.2.3. Based on the docking binding affinities, ursolic acid and andrographolide exhibited the most favorable predicted binding conformations with the binding pockets. Consequently, these top two candidates were selected for further rigorous 100 ns molecular dynamics (MD) simulations using AMBER 24 to computationally assess their dynamic structural stability.

### 3.13. In Silico Molecular Dynamics Simulations Evaluate Predicted Antioxidant Candidates

**Figure 9:**
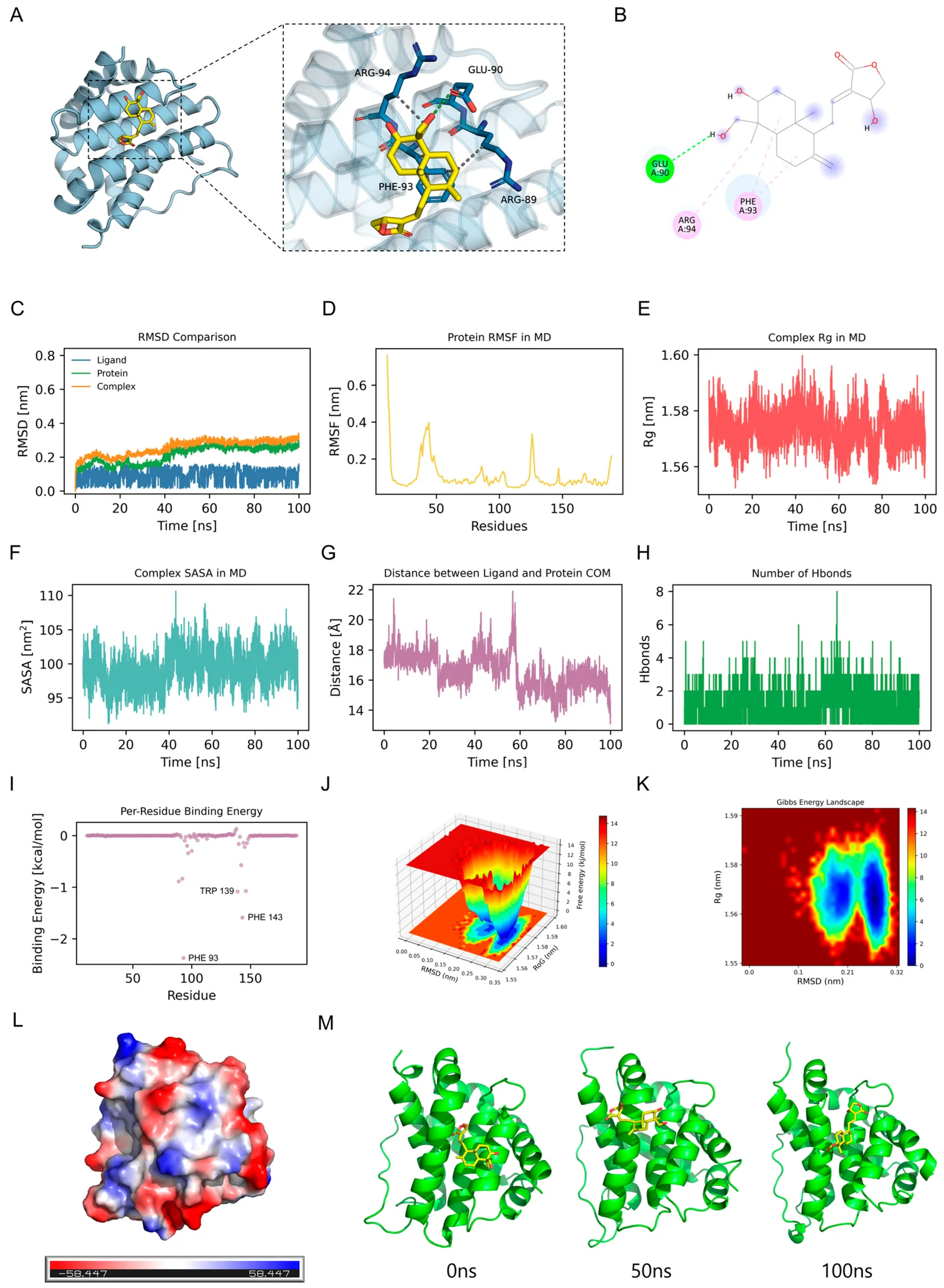
In silico molecular docking and molecular dynamics simulation analysis of the predicted BAX-andrographolide complex. (**A**) The overall binding conformation of the small molecule in the protein binding pocket. The right panel is a local enlarged view of the binding site, displaying the interaction modes between key residues and the ligand. The blue cartoon represents the protein, the yellow sticks represent the ligand, green dashed lines indicate hydrogen bonds, and gray dashed lines indicate hydrophobic interactions; (**B**) a 2D schematic diagram of the interactions between the ligand and key amino acid residues; (**C**) RMSD changes in the ligand, receptor, and complex during the 100 ns molecular dynamics simulation; (**D**) RMSF distribution of the protein backbone residues; (**E**) changes in the radius of gyration (Rg) of the complex during the simulation; (**F**) changes in the solvent-accessible surface area (SASA) of the complex over time; (**G**) changes in the distance between the center of mass (COM) of the ligand and the protein; (**H**) changes in the number of hydrogen bonds between the protein and the ligand during the simulation; (**I**) energy contribution analysis of key amino acid residues to the binding free energy; (**J**) 3D free energy landscape (FEL) constructed based on RMSD and Rg; (**K**) 2D projection map of the free energy landscape; (**L**) electrostatic potential distribution map on the protein surface; (**M**) conformational changes in the complex at 0 ns, 50 ns, and 100 ns during the molecular dynamics simulation.

**Figure 10:**
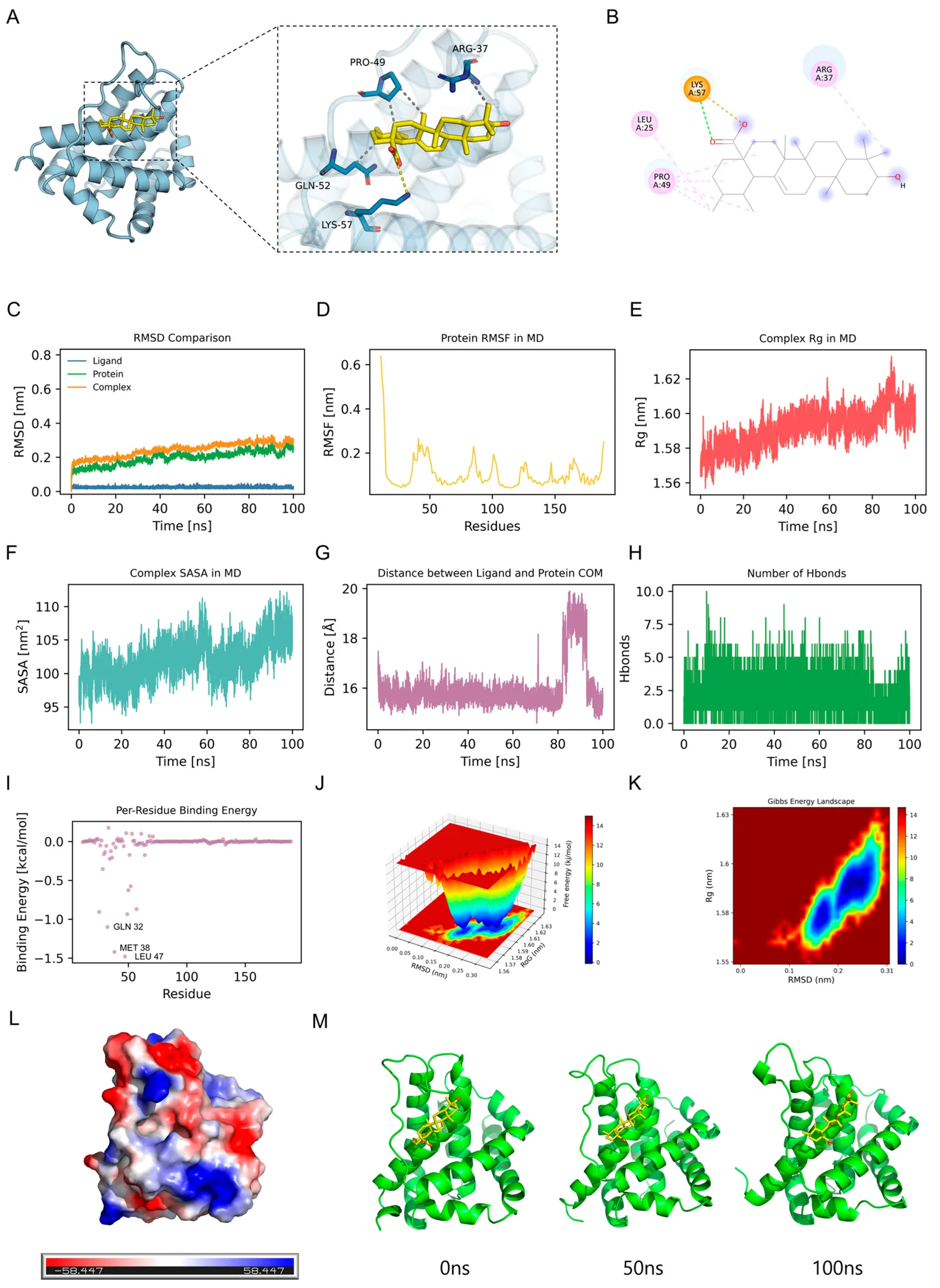
In silico molecular docking and molecular dynamics simulation analysis of the predicted BAX-Ursolic acid complex. (**A**) The overall binding conformation of the small molecule in the protein binding pocket. (**B**) A 2D schematic diagram of the interactions between the ligand and key amino acid residues; (**C**) RMSD changes in the ligand, receptor, and complex during the 100 ns molecular dynamics simulation; (**D**) RMSF distribution of the protein backbone residues; (**E**) changes in the radius of gyration (Rg) of the complex during the simulation; (**F**) changes in the solvent-accessible surface area (SASA) of the complex over time; (**G**) changes in the distance between the center of mass (COM) of the ligand and the protein; (**H**) changes in the number of hydrogen bonds between the protein and the ligand during the simulation; (**I**) energy contribution analysis of key amino acid residues to the binding free energy; (**J**) 3D free energy landscape (FEL) constructed based on RMSD and Rg; (**K**) 2D projection map of the free energy landscape; (**L**) electrostatic potential distribution map on the protein surface; (**M**) conformational changes in the complex at 0 ns, 50 ns, and 100 ns during the molecular dynamics simulation.

**Figure 11:**
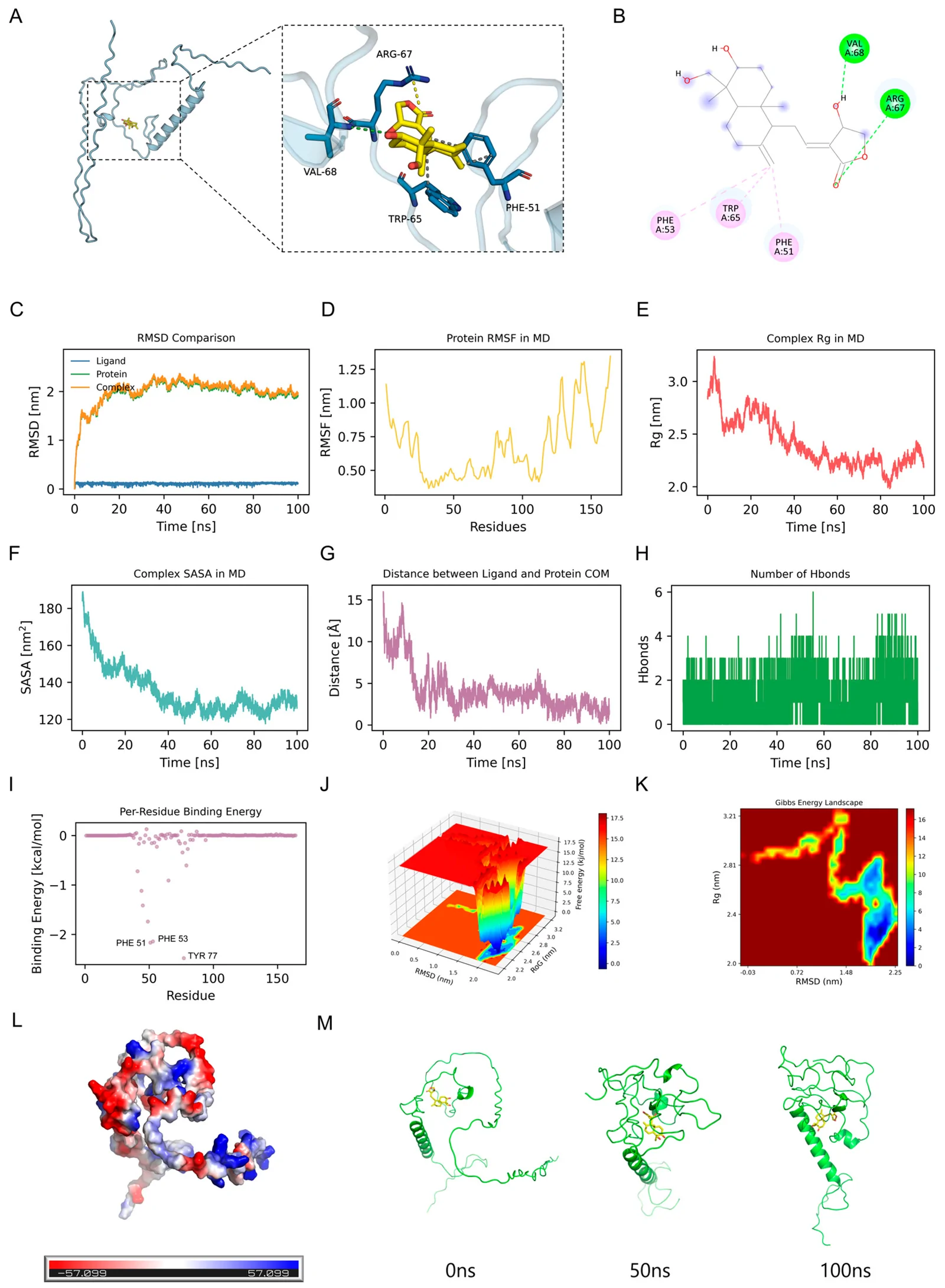
In silico molecular docking and molecular dynamics simulation analysis of the predicted CDKN1A-Andrographolide complex. (**A**) The overall binding conformation of the small molecule in the protein binding pocket. (**B**) A 2D schematic diagram of the interactions between the ligand and key amino acid residues; (**C**) RMSD changes in the ligand, receptor, and complex during the 100 ns molecular dynamics simulation; (**D**) RMSF distribution of the protein backbone residues; (**E**) changes in the radius of gyration (Rg) of the complex during the simulation; (**F**) changes in the solvent-accessible surface area (SASA) of the complex over time; (**G**) changes in the distance between the center of mass (COM) of the ligand and the protein; (**H**) changes in the number of hydrogen bonds between the protein and the ligand during the simulation; (**I**) energy contribution analysis of key amino acid residues to the binding free energy; (**J**) 3D free energy landscape (FEL) constructed based on RMSD and Rg; (**K**) 2D projection map of the free energy landscape; (**L**) electrostatic potential distribution map on the protein surface; (**M**) conformational changes in the complex at 0 ns, 50 ns, and 100 ns during the molecular dynamics simulation.

**Figure 12:**
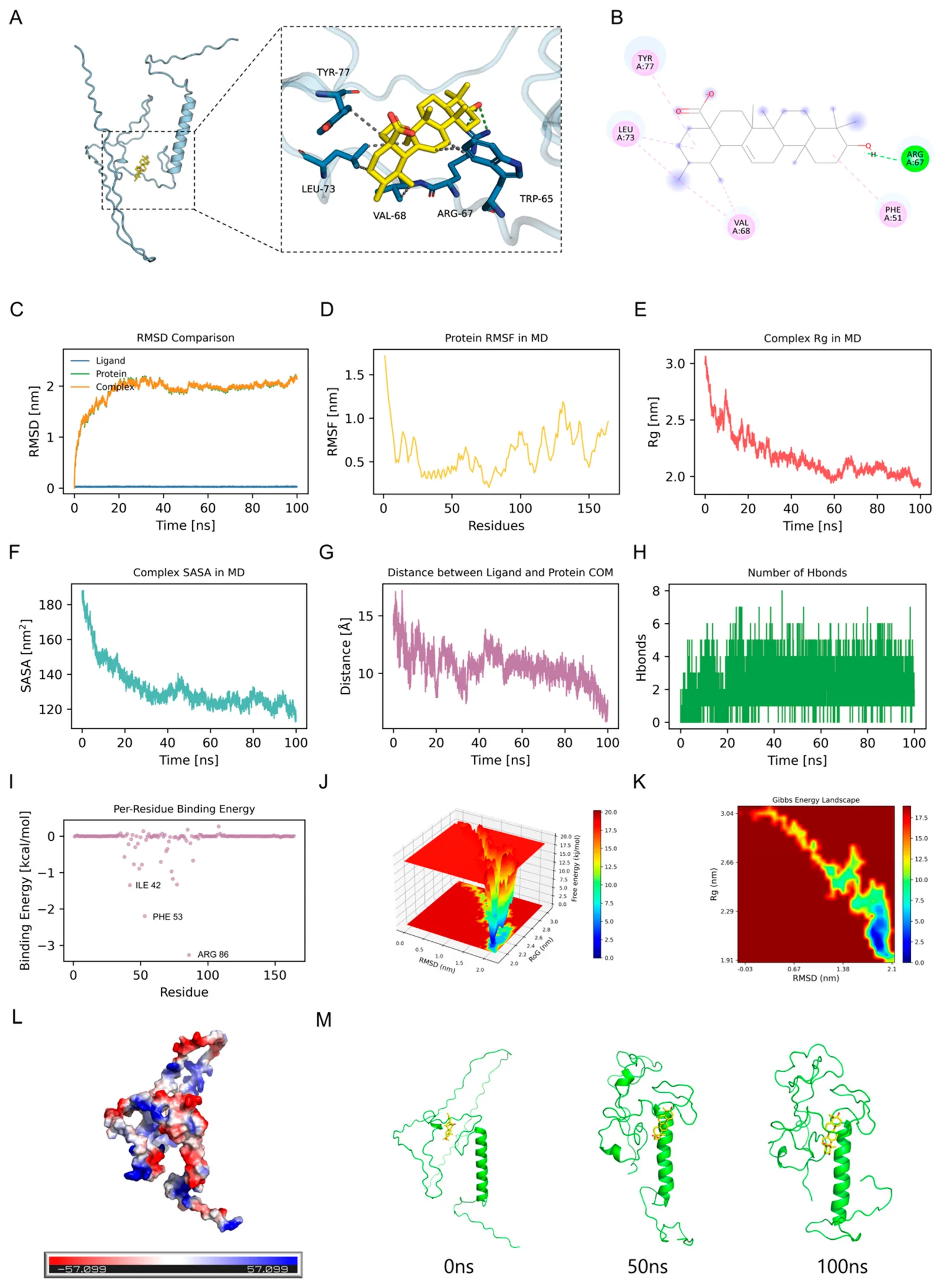
In silico molecular docking and molecular dynamics simulation analysis of the predicted CDKN1A-Ursolic-acid complex. (**A**) The overall binding conformation of the small molecule in the protein binding pocket. (**B**) A 2D schematic diagram of the interactions between the ligand and key amino acid residues; (**C**) RMSD changes in the ligand, receptor, and complex during the 100 ns molecular dynamics simulation; (**D**) RMSF distribution of the protein backbone residues; (**E**) changes in the radius of gyration (Rg) of the complex during the simulation; (**F**) changes in the solvent-accessible surface area (SASA) of the complex over time; (**G**) changes in the distance between the center of mass (COM) of the ligand and the protein; (**H**) changes in the number of hydrogen bonds between the protein and the ligand during the simulation; (**I**) energy contribution analysis of key amino acid residues to the binding free energy; (**J**) 3D free energy landscape (FEL) constructed based on RMSD and Rg; (**K**) 2D projection map of the free energy landscape; (**L**) electrostatic potential distribution map on the protein surface; (**M**) conformational changes in the complex at 0 ns, 50 ns, and 100 ns during the molecular dynamics simulation.

Having experimentally identified the significant transcriptional association of CDKN1A and BAX with SSDE, we utilized computational tools to theoretically explore potential pharmacological interventions directed at these exploratory targets. As detailed in the previous section, following hierarchical database screening, the natural antioxidants ursolic acid and andrographolide emerged as the most promising candidates. We performed molecular docking and 100 ns molecular dynamics (MD) simulations to evaluate their theoretical binding stabilities and dynamic conformational changes. Both compounds exhibited favorable in silico binding affinities with the target proteins. MM/GBSA analysis revealed that ursolic acid strongly bound to CDKN1A and BAX with predicted free energies of −41.10 ± 2.83 kcal/mol and −24.99 ± 2.89 kcal/mol, respectively. Andrographolide also demonstrated stable binding to CDKN1A (−35.56 ± 3.33 kcal/mol) and BAX (−22.62 ± 4.71 kcal/mol) (Table S5). The 3D and 2D interaction models, along with the electrostatic potential maps, showed that robust hydrophobic interactions and polar bonds drive these stable connections (Figure 9A,B,L, Figure 10A,B,L, Figure 11A,B,L and Figure 12A,B,L).

Per-residue energy analysis confirmed that a few specific amino acids in the binding pockets provide the main energy contributions (Figure 9I, Figure 10I, Figure 11I and Figure 12I). The 100 ns MD simulation trajectories supported the structural stability of all complexes. The Root Mean Square Deviation (RMSD) of the ligands remained consistently low, and the structural alignments at 0, 50, and 100 ns showed no signs of dissociation (Figure 9C,M, Figure 10C,M, Figure 11C,M and Figure 12C,M). We closely monitored the Root Mean Square Fluctuation (RMSF), radius of gyration (Rg), and solvent-accessible surface area (SASA). These dynamic metrics indicated that the protein–ligand systems maintained their structural compactness and did not unfold extensively (Figure 9D–F, Figure 10D–F, Figure 11D–F and Figure 12D–F). The consistent distances between the centers of mass (COM) and the continuous hydrogen bond networks further supported a tight binding interface throughout the simulation (Figure 9G,H, Figure 10G,H, Figure 11G,H and Figure 12G,H). The 3D and 2D free energy landscape (FEL) demonstrated that the complexes successfully reached highly stable, low-energy states (Figure 9K, Figure 10K, Figure 11K and Figure 12K).

These comprehensive computational results indicate that ursolic acid and andrographolide exhibited favorable predicted binding conformations within the critical pockets of CDKN1A and BAX, offering a theoretical structural basis to explore the modulation of the oxidative stress–apoptosis cascade in SSDE, which provides an exploratory foundation for future in vitro and in vivo experimental verification.

## 4. Discussion

In this investigation, we successfully isolated 12 core oxidative stress-related differentially expressed genes (OSRDEGs) from the global transcriptomic landscape of the conjunctiva in patients with Sjögren’s syndrome dry eye (SSDE). These genes are potentially implicated in the pathogenesis of SSDE, suggesting exploratory roles in immune dysregulation (TICAM1, CXCL2, ICAM1), apoptosis (BAX, BID), and TGF-β-mediated fibrosis (TGFBR1) [34,35,36,37,38]. Elevated CDKN1A, a classical cell cycle inhibitor, mediates epithelial proliferation arrest—potentially as an initial protective stress response. However, its sustained overexpression, coupled with BAX activation, is hypothesized to drive severe apoptotic damage on the ocular surface [37,39,40]. Meanwhile, TGFBR1 is implicated in SMAD-dependent fibrotic responses, reflecting glandular pathology and dysfunction in secretion through the mislocalization of aquaporin-5 [41,42].

A striking finding in our transcriptome profile is the prominent upregulation of S100A9 in the SSDE conjunctiva. As a central pro-inflammatory marker associated with ocular and systemic autoimmune diseases (e.g., RA, SLE) [43,44,45], S100A9 functions as a potent damage-associated molecular pattern (DAMP) under SSDE-induced stress [46]. Released by damaged epithelial and innate immune cells, it binds to receptors like RAGE and TLR4, triggering inflammatory cascades that amplify cytokine secretion and drive dendritic cell-mediated Th17 responses. Consequently, elevated S100A9 expression reflects an unremitting inflammatory loop and severe epithelial barrier breakdown. The expression profiles of these candidate biomarkers (CDKN1A, BAX, TGFBR1, and S100A9) suggest they might serve as potential exploratory adjuncts to traditional tests like the Schirmer test, which objectively measures tear secretion but does not capture subjective symptom burden [47]. Since SSDE diagnosis is challenged by the symptom-sign discrepancy, integrating these molecular biomarkers with both subjective assessments (e.g., Ocular Surface Disease Index, OSDI) and objective clinical tests could potentially enhance diagnostic precision.

Our external validation using the independent GSE176510 dataset uncovered a robust co-expression relationship between S100A9 and BAX, offering preliminary biological insights into the coordinated progression of SSDE [48]. S100A9, released as an alarmin under stress conditions, acts upstream to engage pattern recognition receptors and amplify inflammatory cascades [49,50]. Concurrently, BAX serves as an essential downstream executioner of the intrinsic apoptotic pathway, permeabilizing the mitochondrial outer membrane [51,52]. The strong positive co-expression of these two factors suggests a synergistic vicious cycle at the ocular surface: sustained inflammatory damage triggered by S100A9 accumulation appears to directly parallel, and potentially accelerate, BAX-mediated mitochondrial apoptotic shedding of conjunctival epithelial cells. This dynamic underscores that ocular surface barrier failure in SSDE is likely governed by an intimately coupled immune–apoptotic axis rather than isolated, independent stress events [48,53].

Beyond core biomarkers, an array of identified hub genes—including *ICAM1*, *CXCL2*, and *IKBKG*—serves as critical bridges linking immune–inflammatory networks to redox imbalance [54,55,56,57]. Crucially, our unbiased global GSEA suggested the profound activation of the KEAP1-NFE2L2 axis alongside NF-κB and MAPK inflammatory cascades. Previous research indicates that signaling axes like NF-κB, MAPK, and NFE2L2 exhibit bidirectional regulatory roles between inflammatory responses and redox homeostasis, suggesting a strong coupling between immune activation and oxidative stress in the pathogenesis of Sjögren’s syndrome-related dry eye [57,58].

Given this autoimmune foundation, CXCL2 and ICAM1 provide valuable candidate clues for exploring specific immune–inflammatory mechanisms underlying SSDE. CXCL2, a ligand for CXCR2, has been shown in experimental inflammation models to participate in neutrophil transendothelial migration, suggesting a potential role in inflammatory cell recruitment; however, its specific mechanisms in SSDE require further clarification [59]. Meanwhile, ICAM1 is fundamentally involved in leukocyte adhesion and intercellular immune interactions. Previous studies have reported increased ICAM1 expression in the conjunctival and accessory lacrimal gland tissues of dry eye patients. Moreover, blocking ICAM1 or LFA-1 has been shown to reduce ocular inflammatory infiltration in Sjögren’s syndrome-like mice, providing supportive experimental evidence for its role in ocular surface immunity [60]. Interestingly, in our present study, while the area under the curve (AUC) values for CXCL2 and ICAM1 were 0.778 and 0.794 in the primary self-test cohort, respectively, they were only 0.429 and 0.684 in the external GSE176510 dataset, indicating significant cohort-dependent variations in their discriminative performance. Taking the existing immunological evidence into account, both molecules remain promising candidate biomarkers for future mechanistic studies focusing on immune cell recruitment and adhesion processes. Nevertheless, their disease-specific pathogenic roles and precise interventional value cannot be definitively established solely based on current expression correlations or ROC analyses. Thus, these core genes reflect the critical but complex crosstalk between oxidative stress and immune–apoptotic injury.

Immune infiltration profiling further highlighted a prominent correlation between activated CD4+ memory T cells and eosinophils (r = 0.843), alongside an inversion between M1 and M2 macrophage subsets (r = −0.704). This imbalance is associated with elevated levels of pro-inflammatory cytokines (IL-6, TNF-α) and serum immunoglobulin G/rheumatoid factor (IgG/RF) [6]. Eosinophil recruitment, which is driven by IL-5, exacerbates the fibrotic process, while the modulation of macrophage polarization presents a promising therapeutic strategy to restore immune tolerance. Importantly, our global pathway enrichment analysis provides an exploratory context for this local immune–apoptotic imbalance. The transcriptomic landscape was overwhelmingly dominated by classical damage pathways, including reactive oxygen species (ROS) accumulation, oxidative phosphorylation, and peroxisome dysfunction. Most notably, the significant enrichment of glycerophospholipid metabolism aligns with our in vivo findings of elevated MDA levels, biochemically suggesting that the conjunctival membrane undergoes severe lipid peroxidation [61]. This profound structural and energetic collapse potentially funnels into apoptotic execution, highlighting an exploratory molecular association, from primary oxidative damage to terminal barrier failure.

Despite these mechanistic insights, targeted pharmacological strategies addressing the underlying oxidative and apoptotic damage in SSDE remain scarce. Expanding upon our bioinformatic and experimental findings, we integrated computer-aided drug discovery (CADD) to predict potential pharmacological interventions. Our 100 ns MD simulations demonstrated, at an atomic resolution, that natural antioxidants—ursolic acid and andrographolide—could theoretically establish stable binding conformations within the active pockets of CDKN1A and BAX. Both ursolic acid and andrographolide have been extensively documented for their potent anti-inflammatory and oxidative stress-alleviating properties in various inflammatory disease models [61,62,63]. By computationally targeting CDKN1A (a cell cycle arrest mediator) and BAX (a pro-apoptotic effector), these compounds are predicted to act as computational interacting ligands, offering a theoretical structural basis to explore the modulation of the aberrant oxidative stress–apoptosis cascade.

Naturally, several limitations of this study should be acknowledged. First, the non-invasive CIC technique yields extremely limited cellular mass, precluding direct protein-level quantification in clinical samples. To offset this physical constraint, we incorporated an in vivo murine SSDE model (NOD vs. BALB/c). However, we explicitly acknowledge that the elevated MDA levels observed in murine lacrimal glands represent cross-species and cross-tissue evidence. While this provides supportive biological context, it does not directly validate the specific oxidative stress status within the human conjunctival epithelium analyzed by our RNA-seq pipeline. Second, the inherent biological heterogeneity of human ocular surface tissues (influenced by unavoidable environmental and lifestyle confounders), combined with the limited sample size restricted by the low RNA yield of non-invasive CIC, constrained our statistical power. Consequently, our transcriptomic analysis relied on nominal *p*-values without surviving strict FDR correction, rendering these transcriptomic pathways strictly exploratory. Third, our ROC and CIBERSORT analyses remain preliminary. The absence of ROC confidence intervals due to the small cohort size, and the reliance on uncorrected correlation testing limit the statistical generalizability of these findings. Finally, the lack of non-SS dry eye disease control groups (e.g., evaporative dry eye) precludes determining whether this exploratory oxidative stress signature is specific to SSDE or a common pathway across dry eye diseases, and our retrospective design did not systematically capture multifactorial clinical variables (e.g., prolonged digital screen use, precise menopausal status, or sleep disorders). Future large-scale, multi-center studies encompassing diverse dry eye subtypes and exhaustive clinical profiling will be vital to validate the diagnostic specificity of these candidate biomarkers.

## 5. Conclusions

In summary, oxidative stress and its coupled transcriptomic signature—anchored by the core candidate biomarkers CDKN1A, BAX, TGFBR1, and S100A9—are potentially implicated in the pathogenesis of SSDE, serving as exploratory diagnostic indicators and potential molecular nodes for future evaluation. Our computational evaluations, including molecular docking and 100 ns MD simulations, suggested that natural antioxidants such as ursolic acid and andrographolide exhibited favorable predicted binding conformations within the functional pockets of CDKN1A and BAX. Together with our clinical transcriptomic data and supportive in vivo biochemical context, these findings provide preliminary mechanistic insights into the interplay between oxidative stress and immune–apoptotic destruction, establishing an exploratory theoretical foundation for future translational and pharmacological investigations.

## Figures and Tables

**Table 1:** Sequences of the primers for real-time PCR.

Gene	Sequences	
*CDKN1A*	Forward Primer	5′-GCCCGTGAGAGATGCCAGA-3′
	Reverse Primer	5′-GGCGCTTGGAGTGATAGAAATC-3′
*BAX*	Forward Primer	5′-CCCGAGCTGTGCCACCAAGA-3′
	Reverse Primer	5′-CCAGCCCATCTTCTTCCAGATGG-3′
*TGFBR1*	Forward Primer	5′-TGGTCAGGAACTGTGCTGAC-3′
	Reverse Primer	5′-CACCAGTCTGGGTCTTGATG-3′
*S100A9*	Forward Primer	5′-AGAATCACCAGCCCCTCTTG-3′
	Reverse Primer	5′-GCCTTGTCACAGGGCACTTA-3′

**Table 2:** Clinical parameters of the total study population (*n* = 31). OSDI, Ocular Surface Disease Index; TBUT, tear film break-up time; SIT, Schirmer I test; CFS, corneal fluorescein staining; SSDE, Sjögren’s syndrome dry eye.

	SSDE	Control	*p*-Value
Number	16	15	-
Gender (Female)	16	15	-
Age (years)	43.88 ± 14.00	40.20 ± 8.41	0.387
OSDI	39.18 ± 21.98	5.91 ± 2.49	<0.001
SIT (mm)	4.25 ± 2.57	19.20 ± 4.55	<0.001
TBUT (seconds)	3.188 ± 1.56	12.53 ± 3.40	<0.001
CFS	3.94 ± 1.53	0.93 ± 0.70	<0.001

**Table 3:** Clinical parameters of the RNA-sequencing subgroup (*n* = 16) OSDI, Ocular Surface Disease Index; TBUT, tear film break-up time; SIT, Schirmer I test; CFS, corneal fluorescein staining; SSDE, Sjögren’s syndrome dry eye.

	SSDE	Control	*p*-Value
Number	9	7	-
Gender (Female)	9	7	-
Age	46.44 ± 15.19	38.86 ± 8.30	0.225
OSDI	40 ± 25.30	5.51 ± 2.50	0.003
SIT (mm)	4.11 ± 2.57	17.43 ± 4.89	<0.001
TBUT (seconds)	2.78 ± 1.30	10.14 ± 2.193	<0.001
CFS	3.22 ± 1.48	1.14 ± 0.69	<0.005

## Data Availability

The RNA-sequencing data generated in this study have been deposited in the NCBI Sequence Read Archive (SRA) under BioProject accession number PRJNA1348748. The public dataset GSE176510 (platform GPL28577) was obtained from the Gene Expression Omnibus (GEO) database and served as an independent external validation cohort. All data are freely available for research purposes.

## Supplementary Materials

The following supporting information can be downloaded at: https://www.mdpi.com/article/10.3390/biomedicines14102277/s1, Table S1: Clinical parameters; Table S2: DEGs; Table S3: OSRG; Table S4: Top 20 candidate drugs targeting the validated hub genes predicted by DSigDB; Table S5: Molecular docking binding affinities of the four screened natural antioxidants against CDKN1A and BAX.
